# Whole genome methylation profiles as independent markers of survival in stage IIIC melanoma patients

**DOI:** 10.1186/1479-5876-10-185

**Published:** 2012-09-05

**Authors:** Luca Sigalotti, Alessia Covre, Elisabetta Fratta, Giulia Parisi, Paolo Sonego, Francesca Colizzi, Sandra Coral, Samuele Massarut, John M Kirkwood, Michele Maio

**Affiliations:** 1 Cancer Bioimmunotherapy Unit, Centro di Riferimento Oncologico, Istituto di Ricovero e Cura a Carattere Scientifico, Aviano, Italy; 2 Division of Medical Oncology and Immunotherapy, Department of Oncology, University Hospital of Siena, Istituto Toscano Tumori, Siena, Italy; 3 CBM scrl - Genomics, Area Science Park, Basovizza, Trieste, Italy; 4 Breast Surgery Unit, Centro di Riferimento Oncologico, Istituto di Ricovero e Cura a Carattere Scientifico, Aviano, Italy; 5 University of Pittsburgh School of Medicine, Pittsburgh, PA, USA

**Keywords:** Whole-genome methylation profiling, Prognosis, Prognostic signature, Hypermethylation, Immunotherapy

## Abstract

**Background:**

The clinical course of cutaneous melanoma (CM) can differ significantly for patients with identical stages of disease, defined clinico-pathologically, and no molecular markers differentiate patients with such a diverse prognosis. This study aimed to define the prognostic value of whole genome DNA methylation profiles in stage III CM.

**Methods:**

Genome-wide methylation profiles were evaluated by the Illumina Human Methylation 27 BeadChip assay in short-term neoplastic cell cultures from 45 stage IIIC CM patients. Unsupervised K-means partitioning clustering was exploited to sort patients into 2 groups based on their methylation profiles. Methylation patterns related to the discovered groups were determined using the nearest shrunken centroid classification algorithm. The impact of genome-wide methylation patterns on overall survival (OS) was assessed using Cox regression and Kaplan-Meier analyses.

**Results:**

Unsupervised K-means partitioning by whole genome methylation profiles identified classes with significantly different OS in stage IIIC CM patients. Patients with a “favorable” methylation profile had increased OS (P = 0.001, log-rank = 10.2) by Kaplan-Meier analysis. Median OS of stage IIIC patients with a “favorable” vs. “unfavorable” methylation profile were 31.5 and 10.4 months, respectively. The 5 year OS for stage IIIC patients with a “favorable” methylation profile was 41.2% as compared to 0% for patients with an “unfavorable” methylation profile. Among the variables examined by multivariate Cox regression analysis, classification defined by methylation profile was the only predictor of OS (Hazard Ratio = 2.41, for “unfavorable” methylation profile; 95% Confidence Interval: 1.02-5.70; *P* = 0.045). A 17 gene methylation signature able to correctly assign prognosis (overall error rate = 0) in stage IIIC patients on the basis of distinct methylation-defined groups was also identified.

**Conclusions:**

A discrete whole-genome methylation signature has been identified as molecular marker of prognosis for stage IIIC CM patients. Its use in daily practice is foreseeable, and promises to refine the comprehensive clinical management of stage III CM patients.

## Background

Cutaneous melanoma (CM) is an aggressive neoplasm with growing incidence and mortality rates in industrialized countries, and the leading cause of skin cancer-related deaths worldwide [1]. At present, the only established prognosticator of 5-year survival, used clinically for routine therapy and for clinical trials, is the clinico-pathological stage of disease. Overall survival (OS) rates range from 95% for stage I to 7% for stage IV patients [2,3]. However, the clinical course of CM within clinico-pathological stages also differs radically, and the lack of prognostic markers has impaired our ability to identify subjects with highly aggressive as opposed to indolent course of disease [4].

Methylation of genomic DNA in mammals occurs at the 5 C-position of cytosine in the context of CpG dinucleotides, resulting in gene silencing through different mechanisms [5]. Alterations in genomic DNA methylation represent a hallmark of cancer and actively contribute to cancer development and progression through inactivation of tumor suppressor genes (TSG) by aberrant promoter hypermethylation [6]. In particular, epigenetic alterations have emerged as important factors in tumor progression in CM, as demonstrated by the growing list of genes that are transcriptionally inactivated by aberrant DNA hypermethylation in this neoplasia, affecting virtually every pathway known to be important for its biology [5]. The increasing role of aberrant methylation in CM biology strongly suggests the potential of methylation markers as indicators of disease prognosis. Along this line, preliminary studies highlight a possible prognostic role of the methylation status of selected genes in CM patients. Among these, *PTEN* methylation emerged as an independent negative prognostic factor in a cohort of 230 patients with stage 0 to IV of disease; however, it did not outperform traditional markers of tumor thickness and ulceration [7]. Similarly, methylation of the TSG *TSLC1* was found to be significantly increased in advancing CM stages, where it was associated with reduced disease-related survival [8]. Furthermore, methylation of the ‘*methylated in tumors*’ (MINT) locus 31 was shown to be significantly associated with advancing clinical stage among 107 stage I to IV CM patients, predicting improved disease-free survival and OS of the 25 stage III patients analyzed [9]. While these data are encouraging, studies reported to date have several limitations, including: i) investigation of only single/few genes; ii); a priori selection of genes to be evaluated; iii) analysis of CM patients populations with highly heterogeneous stages of disease. To address these issues, in an initial search for the prognostic significance of DNA methylation of CM cells, we have recently demonstrated that global genomic DNA hypermethylation, as evaluated on the *Long Interspersed Nucleotide Element-1* (*LINE-1*) repetitive sequences, is associated with significantly reduced OS among stage IIIC CM patients [10].

Based on these promising findings, this study aimed to explore whether whole-genome methylation profiles may account for the differential prognosis of CM patients within an identical clinico-pathological stage of disease. To this end, neoplastic cells from 45 stage IIIC CM patients were analyzed for their genome-wide methylation profiles by the Infinium HumanMethylation27 BeadChips, which allow interrogation of methylation for over 14,000 genes. Autologous short-term neoplastic cell cultures were utilized instead of tumor tissues to overcome alterations in the evaluation of CM-specific genomic methylation profiles due to the presence of contaminating normal cells.

The results generated demonstrate that whole-genome methylation profiling is a powerful tool to identify CM patients with a significantly different prognosis, and that a methylation signature of 17 genes can be utilized to assign CM patients to distinct prognostic groups.

## Methods

### Patients and cell cultures

Short-term cell cultures were established from metastatic lesions removed surgically from consecutive CM patients referred to the National Cancer Institute of Aviano (Italy) for stage III surgery from 1991 to 2007, as previously described [11]. Patients provided written informed consent as per Italian regulations. Success rate in establishing autologous tumor cell cultures from neoplastic tissues was approximately 30%. The micrometastatic nature of lymph-node tumor tissues from AJCC stage IIIA patients precluded their use for cell culture generation, while short-term CM cultures were available from 13 stage IIIB patients, and so were excluded from the study. Thus, the planned investigations were conducted on short-term cultures generated from a total of 45 CM patients, classified as AJCC stage IIIC, who received highly heterogeneous treatments for their disease, including chemotherapy with different agents, immunotherapy, and radiotherapy. Forty-three cell cultures were derived from metastatic lymph-nodes ad 2 from subcutaneous loco-regional lesions. Short-term CM cell cultures were grown in RPMI 1640 Medium supplemented with 20% heat-inactivated fetal calf serum and 2 mM L-glutamine (Biochrome KG, Berlin, Germany). To minimize alterations potentially arising with extended in vitro culturing, all cell cultures were utilized for molecular assays at the 6^th^ ex vivo passage. Cell cultures were confirmed to contain ≥ 95% CM cells as determined by indirect immunofluorescence staining followed by flow cytometric analyses for melanoma-associated antigens (data not shown).

### Whole genome methylation profiling

Genomic DNA was extracted from short-term cultures of CM cells by a standard proteinase K protocol [12]. The 45 samples under analysis were evaluated for genome-wide promoter methylation at the Cluster in Biomedicine scrl (Trieste, Italy) using the Illumina Infinium HumanMethylation27 Bead array (Illumina Inc, San Diego, CA). Analyses were conducted on 500 ng of genomic DNA, and were performed as per Illumina protocol. The arrays were imaged using a BeadArray™ Reader. Image processing and intensity data extraction were performed according to Illumina's instructions. The methylation status of a specifc CpG site was calculated from the intensity of the methylated (M)- and unmethylated (U)-specific beads, as the ratio of fluorescent signals: β = Max(M,0)/[Max(M,0) + Max(U,0)]. DNA methylation β values are continuous variables between 0, no methylation, and 1, completely methylated.

### qMSP analyses

Genomic DNA was subjected to modification with sodium bisulfite using the EZ DNA Methylation-Gold Kit (Zymo Research, Orange, CA, USA). Primers for the analysis of the methylation status of *ALOX12B*, *SLC6A11*, *TUB*, and *WNT10B*, were designed using the free on-line software MethPrimer [13], and are reported in the Table 1. SYBR green qMSP reactions were performed with methylated- or unmethylated-specific primer pairs on 2 μl of bisulfite-modified genomic DNA. The copy number of methylated or unmethylated sequences for each target gene were established by extrapolation from the standard curves. The % of methylation was defined as the ratio between methylated molecules and the sum of methylated and unmethylated molecules.

**Table 1:** Sequences of primers used for qMSP and qRT-PCR assays

**qMSP**		
**GENE**	**Primer sequence**^**1**^	**Amplicon size (bp)**^**2**^
SLC6A11	MF: TGTTTAGGGTTGGGAAGAAGTTAC	128
	MR: ATCGCAATAAACTAAAAAACCTACG	
	UF: TGTTTAGGGTTGGGAAGAAGTTAT	133
	UR: AATAAATCACAATAAACTAAAAAACCTACA	
TUB	MF: TGGTTGTTAGTTTGATTGTTGTTAC^3^	96
	MR: AAAACCTATTAAAATTCCCTATATTCG	
	UF: GTGGTTGTTAGTTTGATTGTTGTTAT	97
	UR: CTAAAACCTATTAAAATTCCCTATATTCA	
ALOX12B	MF: TTCTCTTACCTACCTTAAACCTTCG	152
	MR: TGAGATGGAGTTTCGTGTTTTC	
	UF: TTCTCTTACCTACCTTAAACCTTCA	154
	UR: AGTGAGATGGAGTTTTGTGTTTTT	
WNT10B	MF: TGGGGTGTATAGGTAAAGGTAAATC	91
	MR: GAAAATAAATCAAACGAAAACACG	
	UF: TGGGGTGTATAGGTAAAGGTAAATT	93
	UR: TCAAAAATAAATCAAACAAAAACACA	
**qRT-PCR**		
**GENE**	**Primer sequence**	**Amplicon size (bp)**
ALOX12B	F: ACCCGAGGGCAAGATGAT	74
	R: GCAGGAAGATGGGGCAAT	
SLC6A11	F: AGGGGGTACCCATTGCTG	65
	R: CTTGGGGTACGCAATAAAGG	
TUB	F: TCCGACTGGATTCCCTACAG	109
	R: GGCGCTTCTTCTTCTGCTT	
WNT10B	F: GCGAATCCACAACAACAGG	107
	R: TCCAGCATGTCTTGAACTGG	

^1^*MF*, methylated forward primer; *MR*, methylated reverse primer; *UF*, unmethylated forward primer; *UR*, unmethylated reverse primer; *F*, forward primer; *R*, reverse primer.

^2^ Size of the amplification product in base pairs.

^3^ Underlined text represent locked nucleic acid (LNA)-modified nucleotides.

### qRT-PCR analyses

Real-time qRT-PCR analyses were performed as previously described [12]. Primers sets used are listed in Table 1. The copy number of target genes and of the reference gene β-actin were established in each sample by extrapolation from the standard curves. The number of target gene cDNA molecules in each sample was normalized to the number of cDNA molecules of β-actin.

### Statistical analysis

Statistical analyses were performed using the R statistical environment ( http://www.R-project.org) and bioconductor packages ( http://www.bioconductor.org). We used the methylumi package for importing and pre-processing the methylation data, the survival package for Kaplan-Meier estimates, and the pamr package for the shrunken centroid supervised analysis. The methylation data from the Illumina Beadstudio software were imported in R, checked for quality and normalized. K-means partitioning clustering was used to divide the data into 2 groups. The primary objective was to determine differences in OS between the patient groups defined by K-means partitioning. Survival time was calculated in months from the date of stage IIIC diagnosis until the date of death. According with the specific goals of the analysis, we did not classify the deaths considering their cause. Patients were censored at the last follow-up date or the last date the patient was known to be alive. Median survival duration was determined by the Kaplan-Meier method [14]. Cumulative survival was evaluated using the log-rank test. Cox proportional hazard method [15] was used to examine the effect of classification by genome-wide methylation profiles on survival and results were presented as HR with corresponding 95% CI. Variables significantly associated with OS in univariate analysis were included in a multivariate model.

Methylation patterns (signatures) of the discovered groups were determined using the nearest shrunken centroid classification algorithm (PAM). The threshold for balancing between the number of sample correctly classified and the subset of features representing the methylation patterns was determined by cross-validation [16]. The correlation between methylation values from Illumina Infinium and qMSP assays, as well as between gene methylation and mRNA expression, were evaluated by the Spearman’s rank correlation test. The genome-wide methylation profile of each patient was also summarized with a “methylation score” as follows: methylation for each gene among the patients was standardized by the Z score method, each patient was then assigned a “methylation score” consisting of the average of Z scores of all genes. Differences in “methylation score” between k-means defined patient groups were evaluated by Student’s *T* test. Reported P values are two sided and values <0.05 were considered to be statistically significant.

## Results

### Patients

The study was conducted on CM patients who underwent radical lymph node dissection for stage III disease at the Centro di Riferimento Oncologico National Cancer Institute between 1991 and 2007. Patients diagnosed with a stage IIIC disease, and for whom a short-term cell culture had been successfully generated from the surgically removed autologous neoplastic tissue, were included. Table 2 summarizes the 45 patients under study and their clinico-pathologic characteristics at presentation.

**Table 2:** Characteristics of the 45 AJCC stage IIIC melanoma patients

**Variable**	**n. patients**	**%**
Age, years		
Median	52	
Range	29-83	
Gender		
Male	28	62.2
Female	17	37.8
Localization of primary tumor		
extremities	16	35.6
trunk	24	53.3
head & neck	2	4.4
NA^1^	3	6.7
Breslow thickness of primary tumor		
≤2.0 mm	13	28.9
>2.0 mm	25	55.6
NA	7	15.6
Clark level of primary tumor		
1-3	13	28.9
4-5	26	57.8
NA	6	13.3
Ulceration of primary tumor		
No	10	22.2
Yes	33	73.3
NA	2	4.4
N. lymph nodes involved		
1	9	20
>1	35	77.8
NA	1	2.2
LDH^2^		
Low^3^	31	68.9
High	11	24.4
NA	3	6.7
BRAF^V600E^		
mutated	25	55.6
wild-type	15	33.3
NA	5	11.1
*LINE-1*^*4*^		
hypomethylated	22	49
hypermethylated	23	51

^1^ NA, not available.

^2^ LDH, lactate dehydrogenase.

^3^ low LDH is established as LDH values ≤ 0.8 times the upper limit of normal; high LDH is defined as LDH values > 0.8 times the upper limit of normal.

^4^ extent of methylation of *LINE-1* was evaluated by pyrosequencing and defined as hypomethylated or hypermethylated depending on the methylation level being < or ≥ the median of the population, respectively.

### Unsupervised analysis of whole-genome methylation profiles and survival analysis

**Figure 1:**
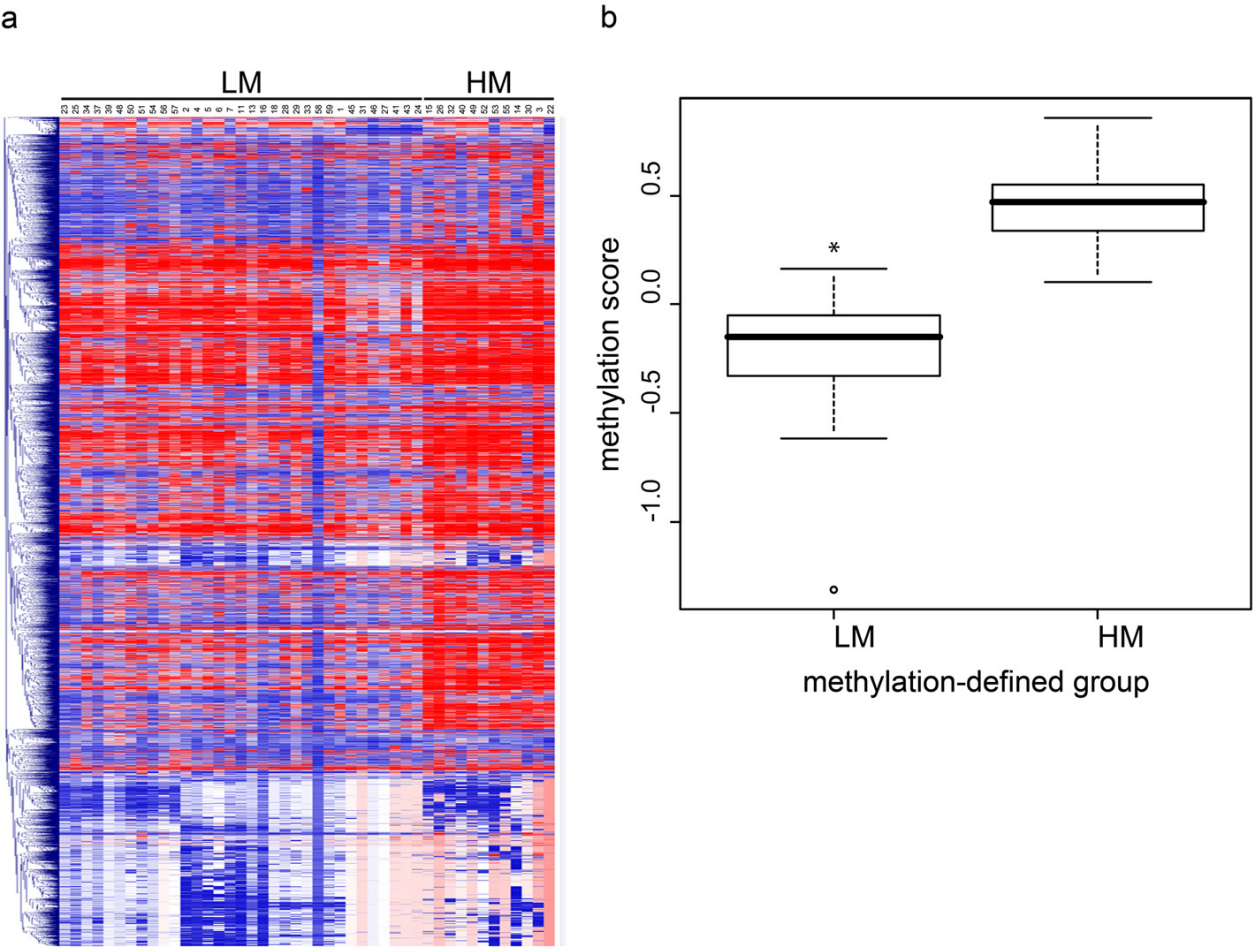
**Groups of stage IIIC CM patients identified by whole-genome methylation profiling.** Genome-wide DNA methylation profile was evaluated by Illumina HumanMethylation27 Bead-Chip whole-genome assay in short-term cultures of CM cells generated from neoplastic lesions of 45 stage IIIC CM patients. Cells were analyzed at 6^th^ in vitro passage. Patients were divided into 2 classes (LM and HM) based on the whole genome methylation profile of their tumor cells through the k-means algorithm. Panel **a**, the whole genome methylation profiles in the patients’ population have been reported as a heatmap. Patient identifier numbers and the k-means-defined groups have been reported on top of the heatmap. Each color patch represents the methylation level of one gene in each patient, with a continuum of methylation levels from dark blue (completely unmethylated, β = 0) to dark red (completely methylated, β = 1). Panel **b**, the genome-wide methylation profile of each patient was summarized by the “methylation score”, where “methylation scores” > and < 0 represent methylation above and below the population’s mean, respectively. Separate box plots have been generated for the LM and HM whole methylome defined patients’ groups. Black horizontal bars represent the median values of “methylation score” for each group. *, P = 0.001 as evaluated by Student’s *T* test.

Genome-wide gene methylation profiles were evaluated in the 45 short-term CM cell cultures under study using the Illumina HumanMethylation27 Bead-Chip whole-genome assay, which interrogates 27,578 CpG sites, corresponding to 14,495 genes. Patients were divided into 2 subgroups, according to their whole genome methylation profile, by the k-means clustering method (Figure 1). The subgroups generated included 33 and 12 patients (Figure 1).

“Methylation scores” were calculated for each patient describing the methylation density of the genome, with values > 0 representing methylation above the population’s mean. Noteworthy, the 2 k-means-defined groups differed significantly (p < 0.001) in their global levels of gene methylation, the mean “methylation score” being −0.2 for the 33 patient group as compared to 0.46 for the 12 patient group (Figure 1). Accordingly, groups were referred to as low-methylation (LM) and high-methylation (HM), respectively.

**Figure 2:**
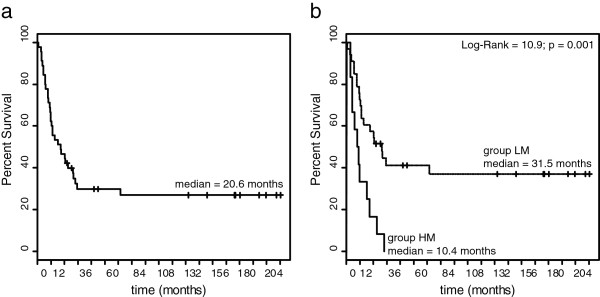
**Kaplan-Meier analysis of stage IIIC CM patients survival according to genome-wide methylation profiles.** Genome-wide DNA methylation profile was evaluated by Illumina HumanMethylation27 Bead-Chip whole-genome assay in short-term cultures of CM cells generated from neoplastic lesions of 45 stage IIIC CM patients. Cells were analyzed at 6^th^ in vitro passage. Patients were divided into 2 classes (LM and HM) based on the whole genome methylation profile of their tumor cells through the k-means algorithm. Kaplan-Meier function for OS was calculated for stage IIIC CM patients either unstratified (**a**), or stratified according to k-means-defined methylation classes (**b**). Dashed and solid lines refer to patients’ groups LM and HM, respectively. Vertical bars in the Kaplan-Meier curves represent censored patients. Cumulative survival by k-means-defined methylation group was evaluated using the Log-Rank test, reported P values were two sided.

The impact of the genome-wide methylation profile-based classification on OS was assessed by Kaplan-Meier analysis. Results demonstrated a significant survival advantage for patients classified as LM as compared to HM (P = 0.001, log-rank = 10.9), with an increased median OS of 31.5 months for LM (95% Confidence Interval (CI): 13.12-inf) compared to 10.4 months (95% CI: 5.29-inf) for HM patients (Figure 2). The 5 year OS of patients classified as LM and HM were 41.2% and 0%, respectively.

Cox univariate analysis was carried out to identify patient characteristics and clinico-pathologic factors that predict OS. Among the range of factors examined, including age, gender, localization, Breslow thickness, Clark level and ulceration of primary tumor, as well as number of lymph nodes involved, pre-operative lactate dehydrogenase, BRAF^V600E^ mutation status, and *LINE-1* methylation, only classification by the genome-wide methylation profiles (Hazard Ratio (HR) =3.3 for group HM vs. LM; 95% CI: 1.56-6.99; P=0.001; Table 3) and *LINE-1* methylation (HR = 2.52 for *LINE-1* hypermethylated vs hypomethylated group; 95% CI: 1.21-5.26; P=0.01; Table 3) were associated with statistically significant differences in OS. When these 2 variables were included in a multivariate Cox model, only whole-methylome defined K-means classification retained a significant impact on OS (Table 3).

**Table 3:** Univariate and multivariate analysis of the influence of genome-wide methylation profiles on OS of stage III CM patients

		**Univariate**			**Multivariate**		
**Group**^**1**^	**# events/# patients**^**2**^	**HR**^**3**^	**95% CI**	***P***	**HR**^**4**^	**95% CI**	***P***
LM	20/33	1^5^			1		
HM	12/12	3.30	1.56-6.99	0.001	2.41	1.02-5.70	0.045
*LINE-1* hypomethylated	12/22	1			1		
*LINE-1* hypermethylated	20/23	2.52	1.21-5.26	0.01	1.76	0.75-4.13	0.20

^1^ Patients were divided by the k-means clustering algorithm in 2 groups (LM, HM), according to their genome-wide methylation profile, or by *LINE-1* methylation being < (hypomethylated) or ≥ (hypermethylated) the median value of the population;

^2^ number of patients who died (# events) and total number of patients in the group (# patients) are reported.

^3^ Cox proportional hazard method was used to evaluate the effect of the examined variables on OS. Results were presented as Hazard Ratios (HR) with corresponding 95% Confidence Intervals (CI);

^4^ a multivariate Cox proportional hazard model was constructed for OS, including k-means clustering by whole-genome methylation profile and *LINE-1* methylation;

^5^ set as reference.

Despite the above reported impact on OS, χ squared analyses did not show any significant association between methylation profiles and metastatic patterns, with a particular focus on the development of brain metastasis (p = 0.29).

### Identification of the methylation signature of CM patient subgroups with different prognosis

**Figure 3:**
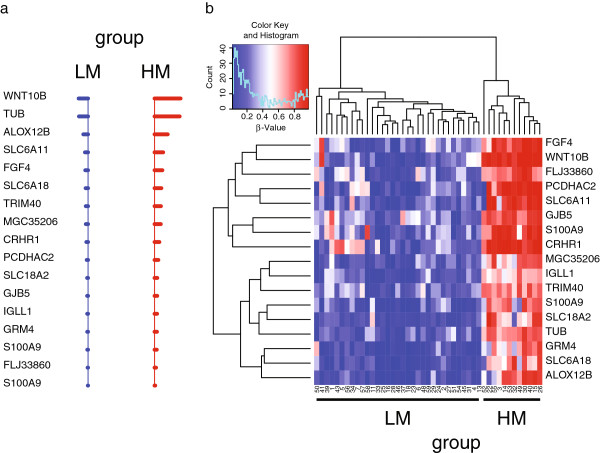
**Prognostic methylation signatures identified by the “nearest shrunken centroid” algorithm in stage IIIC CM patients.** Pre-processed genome-wide methylation data from stage IIIC CM patients under study was analyzed through nearest shrunken centroids algorithm to identify genes best characterizing the k-means defined prognostic methylation groups. Panel **a**, Shrunken differences for the 17 methylation markers, having at least 1 non-zero difference, selected as signature for LM (blue) and HM (red) groups. Lines to the left indicate relatively reduced levels of methylation; lines to the right indicate relatively increased levels of methylation. Genes defining the signature have been reported as Gene Symbol on the left of the panel. Duplicate Gene Symbol entries refer to methylation data read for the same gene from different probes. Panel **b**, the methylation values of the 17 genes composing the methylation signature in the patients’ population have been reported as a heatmap. Genes defining the signature have been reported as Gene Symbol on the right of the heatmap, while patient identifier numbers and the k-means-defined groups have been reported on foot of the heatmap. Duplicate Gene Symbol entries refer to methylation data read for the same gene from different probes. Each color patch represents the methylation level of one gene in each patient, with a continuum of methylation levels from dark blue (completely unmethylated, β = 0) to dark red (completely methylated, β = 1).

To define the methylation signature representing the minimal number of methylation markers characterizing LM and HM patient groups, we applied the “nearest shrunken centroid” algorithm [16] (Figure 3). A methylation signature of 17 genes (Figure 3, Table 4), that allowed to correctly sort stage IIIC patients into groups LM and HM (overall error rate = 0), was identified. A dramatic difference in the methylation status of the genes included in the signature is evident between the good prognosis LM and the bad prognosis HM groups of patients (Figure 3).

**Table 4:** Genes composing the prognostic methylation signature for the 45 stage IIIC CM patients

**TargetID**^**1**^	**GENE ID**^**2**^	**SYMBOL**^**3**^	**Distance to TSS**^**4**^	**CPG ISLAND**^**5**^
cg00498305	6571	SLC18A2	480	TRUE
cg01333788	2709	GJB5	93	FALSE
cg02064402	348932	SLC6A18	249	FALSE
cg02415431	3543	IGLL1	760	FALSE
cg03742272	242	ALOX12B	316	FALSE
cg05164634	7480	WNT10B	995	TRUE
cg05492113	7275	TUB	152	FALSE
cg07039113	6280	S100A9	1317	FALSE
cg08929103	1394	CRHR1	1331	FALSE
cg09196959	135644	TRIM40	70	FALSE
cg09395732	6538	SLC6A11	461	TRUE
cg14236389	284756	FLJ33860	58	TRUE
cg14578030	2249	FGF4	1212	FALSE
cg16139316	6280	S100A9	428	FALSE
cg22088368	339669	MGC35206	1049	FALSE
cg24076884	56134	PCDHAC2	569	TRUE
cg26424956	2914	GRM4	83	FALSE

^1^ Illumina unique probe identifier;

^2^ Unique NCBI's Entrez Gene record (gene identifier) for the gene;

^3^ unique gene symbol as established by the HUGO Gene Nomenclature Committee;

^4^ distance in base pairs from the putative transcription start site of the gene;

^5^ location of the assayed CpG site in a region fulfilling the definition of CpG island.

### Validation of microarray data

**Figure 4:**
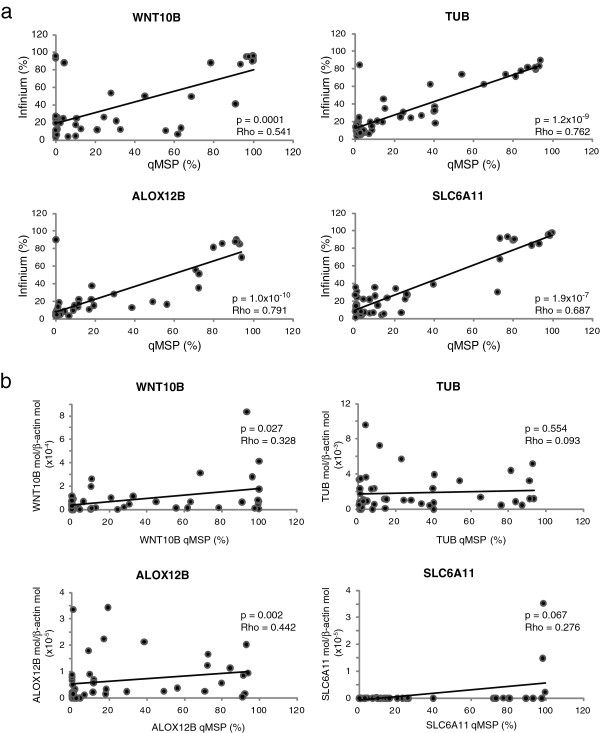
**Validation of microarray data. ** Short-term cultures of CM cells generated from neoplastic lesions of 45 stage IIIC melanoma patients were evaluated for WNT10B, TUB, ALOX12B and SLC6A11 methylation and mRNA expression by qMSP and qRT-PCR analyses, respectively. All cells were analyzed at 6^th^ in vitro passage. For each gene, the % of methylation measured by qMSP was defined as the ratio between methylated gene molecules and the sum of methylated and unmethylated gene molecules. Level of gene expression is reported as number of molecules of the target gene normalized to the number of molecules of the housekeeping gene β-actin. Panel **a**, Correlation between % methylation defined by qMSP and β-values defined by the Illumina HumanMethylation27 Bead-Chip assay was evaluated for each gene through the Spearman’s rank correlation test. Reported P values are two sided. Panel **b**, Correlation between methylation and mRNA expression was evaluated for each gene by Spearman’s rank correlation test. Reported P values are two sided.

Array-based methylation profiling was validated on the short-term CM cell cultures under analysis using quantitative Methylation-Specific PCR (qMSP) for selected genes composing the 17-gene methylation signature. Genes were selected among those having the highest impact on the classification task, which could be identified by their positioning at the top of the shrunken centroids graph (Figure 3a). Spearman’s rank correlation identified a highly significant (p ≤ 10^-4^) positive correlation between methylation values determined by the Illumina Infinium platform and those defined by qMSP, for all of the 4 genes under analysis (*WNT10B*, *TUB*, *ALOX12B*, *SLC6A11*; Figure 4). Coefficients of correlation ranged from 0.541 to 0.791, for *WNT10B* and *ALOX12B*, respectively (Figure 4).

To confirm the tumor-specificity of the methylation patterns identified, qMSP assays were also performed on commercially available epidermal melanocytes and normal peripheral blood mononuclear cells from healthy subjects and selected patients under study. Results demonstrated an invariably unmethylated (*WNT10B*) or methylated (*TUB, ALOX12B, SLC6A1*1) status of the normal samples (data not shown) as compared to the variable levels of methylation observed in tumor cells (Figure 4).

### Correlation with gene expression

The differential gene methylation patterns observed among CM patients could contribute to differential survival through altered expression of the respective genes. To initially evaluate this aspect, the expression of selected genes included in the 17-gene methylation signature was evaluated by quantitative RT-PCR (qRT-PCR) in the 45 short-term CM cell cultures under study. Low, and somewhat heterogeneous levels of WNT10B, TUB, ALOX12B, and SLC6A11 mRNA were observed (Figure 4). A significant (p < 0.05) correlation was found between expression and gene methylation for WNT10B and ALOX12B, though Rho values were < 0.5.

## Discussion

In this study we demonstrate that the genome-wide DNA methylation profile of tumor cells from CM patients with nodal metastases is a significant predictor of OS within stage IIIC. This finding provides the first evidence that genome-wide methylation profiles can serve as molecular markers of prognosis for CM patients in this high-risk group, which has been the focus of multiple adjuvant trials.

Promoter hypermethylation has been proposed to have an important impact on tumor biology through the silencing of TSG and the alteration of virtually every cellular pathway relevant to CM development and progression [5]. Accordingly, initial studies evaluated the status of specific genes known to be methylated in cancer, and showed an association of promoter hypermethylation and advancing tumor stage, reduced disease-related survival and/or OS in CM patients [7,8]. These studies relied on the a priori selection of few genes and generally enrolled CM patients from highly heterogeneous disease stage-groupings.

The results of the present study demonstrate the adverse prognostic impact of genome-wide hypermethylated profiles in relation to OS, and more importantly, for patients within a single stage sub-grouping of disease, have shown remarkable prognostic significance for the methylation profile. This observation strongly supports the notion that the constitutive methylation profile of cancer cells is intimately linked to the behavior of the tumor, and drives differing outcomes of disease. This concept is further strengthened by the discovery that, among all clinico-pathological and molecular factors examined, only those linked to genome-wide methylation were significantly associated with OS among CM patients analyzed. In this context, the multivariate Cox model identified whole-methylome-profile-defined classification as the most robust prognostic marker, suggesting its superior ability in the identification of biologically relevant methylation backgrounds as compared to *LINE-1* methylation [10].

Genome-wide methylation profiles could be recapitulated using a 17-gene signature, which was sufficient to correctly assign CM patients to the identified prognostic groups. Intriguingly, none of the genes composing the signature has been previously reported to be methylated in CM, demonstrating that “unbiased” methylation profiling represents an appropriate and possibly more effective tool for the identification of novel prognostic/predictive epigenetic markers in human cancer.

The mechanisms through which the different methylome profiles affect the survival of CM patients remain to be defined. A direct contribution of the products of the 17 signature genes is unlikely since no meaningful association was observed between methylation and expression of analyzed genes (Figure 4). The methylation signature, thus, appears as an effective *bona fide* prognostic marker, accounting for the overall methylation profile of tumor cells, without a direct impact on tumor biology that can presently be defined. A similar observation was recently reported by Tanemura et al. [9] who evidenced a significant positive association of *MINT31* hypermethylation and improved disease-free survival and OS in stage III CM patients. Since no established product is known for *MINT31* locus, the authors suggested that the methylation status of the locus could be linked to the activation status of additional genes yet to be identified [9]. This appears to be true, more in general, for the widely described phenomenon of the CpG island methylator phenotype (CIMP), which has been described in several tumor types, and refers to a high frequency of concomitant aberrant hypermethylation of different genes and/or chromosomal loci [17]. Indeed, presence of CIMP, rather than accounting for the transcriptional suppression of the specific genes tested, identifies tumors that have a higher propensity to manifest genome-wide hypermethylation, and thus are more likely to inactivate genes critical for tumor progression and response to therapy, leading to a worst prognosis [18]. In line with this notion, functional enrichment analysis of the genes that were significantly differentially methylated between LM and HM groups revealed a perturbation of several biological pathways, including cytokine signaling, cell adhesion, drug and retinol metabolism, and natural killer cell mediated cytotoxicity (Additional file 1). Thus, the global alteration of these pathways could account for the different OS of CM patients bearing different methylomes. Intriguingly, biological processes involved in immune response are highly represented in the genes differentially methylated between HM and LM patients (Additional file 2), suggesting that an improved immune recognition of CM cells with a LM profile might contribute to the better survival of these patients. Though this hypothesis has still to be demonstrated and is currently under investigation, an initial support may come from the well known involvement of promoter methylation in regulating the expression of different molecules involved in the immune recognition of cancer cells, including: i) the *de novo* expression of the Cancer Testis Antigens (CTA) tumor associated antigens by neoplastic cells of different histotype and melanoma stem cells following promoter hypomethylation; ii) the direct correlation between levels and total number of CTA expressed in short-term cultures of CM cells and *LINE-1* hypomethylation (Sigalotti and Maio, unpublished); iii) the ability of pharmacologic DNA hypomethylation to increase immunogenicity and immune recognition of cancer cells through the up-regulation of HLA class I and co-stimulatory/accessory molecules [5,19].

Irrespective of the underlying biological features associated with the different whole-genome methylation profiles, the prognostic value of methylome classification here identified for stage IIIC CM patients bears several important practical clinical implications. Among these are: i) providing an improved clinico-pathological sub-staging; ii) modulating post-surgery follow-up-procedures; iii) selecting patients at higher risk of recurrence for adjuvant treatment(s); iv) stratifying patients in clinical trials based on their differential prognosis. This latter aspect is of particular relevance also in view of multiple studies that have explored new adjuvant regimens in stage III CM patients, in the US and European cooperative groups. These considerations may not be restricted to CM. Indeed, a recent work, investigating whole-genome methylation profiles in breast cancer, demonstrated that the group of patients with high-methylation tumors disproportionately included individuals with a poor prognosis defined by the 70-gene expression prognostic signature of van’t Veer et al. [20].

## Conclusions

In conclusion, the data reported in this study present the first whole-methylome defined prognostic classifier for advanced operable melanoma of stage IIIC, and suggest the evaluation of this for the routine clinico-pathological ascertainment of patients to allow a more accurate assessment of clinical trial results, as well as ultimately to enable personalized management of patients in the clinical routine. Whether the methylation classifier presented in this study will be more easily and effectively translated into the daily clinical practice than previously identified gene expression- and microRNA-based prognostic classifiers [21] is the object of further investigations. Along this line, we are currently planning a prospective study to independently validate our findings and provide the required support for their feasible transfer into the clinical setting. Concomitantly, the pathways affected by aberrant methylation are being carefully explored and functionally characterized to possibly provide new therapeutic targets that could be hit by specific therapeutics, possibly combined with epigenetic treatments.

## Abbreviations

CI: Confidence Intervals; CM: cutaneous melanoma; HM: high-methylation; HR: Hazard Ratio; LDH: lactate dehydrogenase; *LINE-1*: Long Interspersed Nucleotide Element-1; LM: low-methylation; MINT31: Methylated IN Tumors locus 31; OS: overall survival; qMSP: quantitative Methylation-Specific PCR; qRT-PCR: quantitative RT-PCR; TSG: tumor suppressor genes.

## Competing interests

LS and MM have applied for a patent based on the findings reported in this manuscript. All other authors declare no conflict of interest.

## Authors’ contributions

LS participated in designing and coordinating the study, acquiring laboratory data, data analysis and interpretation, and drafted the manuscript. AC, EF, GP, FC contributed in cellular biology procedures, molecular assays and data acquisition and analysis. PS performed the statistical analyses. SC, contributed in data interpretation. SM participated in acquisition of clinical data and data interpretation. JMK participated in data interpretation and manuscript drafting. MM conceived of the study, participated in its design and coordination, and contributed in producing the final draft of the manuscript. All authors read and approved the final manuscript.

## Supplementary Material

Additional file 1 Biological pathways significantly over-represented in genes differentially methylated between HM and LM CM patients. Click here for file

Additional file 2 Biological processes significantly over-represented in genes differentially methylated between HM and LM CM patients. Click here for file
