# Supplementary material for: Whole genome methylation profiles as independent markers of survival in stage IIIC melanoma patients
Source: J Transl Med. 2012 Sep 5;10:185. doi: 10.1186/1479-5876-10-185 (PMC3539917; doi:10.1186/1479-5876-10-185)
Supplement: Additional file 1 — Biological pathways significantly over-represented in genes differentially methylated between HM and LM CM patients. [file 1479-5876-10-185-S1.pdf]

Additional file 1. Biological pathways significantly over-represented in genes differentially methylated between HM and LM CM patients

**KEGG pathway**	**KEGGID**[1]	**P-value**
Cytokine-cytokine receptor interaction	4060	1.32E-16
Neuroactive ligand-receptor interaction	4080	3.12E-14
Hematopoietic cell lineage	4640	1.04E-09
Autoimmune thyroid disease	5320	4.39E-07
Retinol metabolism	830	1.25E-06
Linoleic acid metabolism	591	1.31E-06
Drug metabolism - cytochrome P450	982	9.22E-06
Fat digestion and absorption	4975	1.27E-05
Olfactory transduction	4740	1.52E-05
Staphylococcus aureus infection	5150	0.00016
Steroid hormone biosynthesis	140	0.000227
Drug metabolism - other enzymes	983	0.000253
Malaria	5144	0.000319
Caffeine metabolism	232	0.000378
Cell adhesion molecules (CAMs)	4514	0.000481
Asthma	5310	0.000611
Rheumatoid arthritis	5323	0.000718
Pancreatic secretion	4972	0.000801
Natural killer cell mediated cytotoxicity	4650	0.000821

[1] univocal KEGG pathway identifier
