# Supplementary material for: Whole genome methylation profiles as independent markers of survival in stage IIIC melanoma patients
Source: J Transl Med. 2012 Sep 5;10:185. doi: 10.1186/1479-5876-10-185 (PMC3539917; doi:10.1186/1479-5876-10-185)
Supplement: Additional file 2 — Biological processes significantly over-represented in genes differentially methylated between HM and LM CM patients. [file 1479-5876-10-185-S2.pdf]

Additional file 2. Biological processes significantly over-represented in genes differentially methylated between HM and LM CM patients

**Biological Process**	**GOBPID**[1]	**P-value**
system process	GO:0003008	7.32E-16
sensory perception	GO:0007600	9.59E-16
ion transport	GO:0006811	6.02E-11
cell-cell signaling	GO:0007267	2.23E-10
**immune response**[2]	**GO:0006955**	**5.85E-10**
homeostatic process	GO:0042592	9.94E-09
cellular calcium ion homeostasis	GO:0006874	1.31E-08
ion homeostasis	GO:0050801	1.71E-08
response to other organism	GO:0051707	1.75E-08
**inflammatory response**	**GO:0006954**	**3.08E-08**
G-protein coupled receptor protein signaling pathway	GO:0007186	5.06E-08
**defense response to bacterium**	**GO:0042742**	**5.46E-08**
sensory perception of smell	GO:0007608	8.33E-08
metal ion homeostasis	GO:0055065	8.36E-08
epidermal cell differentiation	GO:0009913	1.14E-07
cellular chemical homeostasis	GO:0055082	3.08E-07
digestion	GO:0007586	3.69E-07
cellular cation homeostasis	GO:0030003	3.95E-07
cellular defense response	GO:0006968	3.95E-07
divalent inorganic cation homeostasis	GO:0072507	4.52E-07
muscle system process	GO:0003012	5.56E-07
muscle filament sliding	GO:0030049	1.06E-06
actin-mediated cell contraction	GO:0070252	1.06E-06
keratinization	GO:0031424	1.64E-06
detection of stimulus	GO:0051606	1.75E-06
positive regulation of cell proliferation	GO:0008284	2.16E-06
elevation of cytosolic calcium ion concentration	GO:0007204	3.15E-06
visual perception	GO:0007601	3.73E-06
epidermis development	GO:0008544	5.11E-06
excretion	GO:0007588	5.33E-06
xenobiotic metabolic process	GO:0006805	7.03E-06
response to xenobiotic stimulus	GO:0009410	8.93E-06
epithelial cell differentiation	GO:0030855	9.63E-06
biological adhesion	GO:0022610	1.22E-05
regulation of response to external stimulus	GO:0032101	1.56E-05
synaptic transmission	GO:0007268	1.84E-05
multicellular organismal process	GO:0032501	1.94E-05
response to stimulus	GO:0050896	2.05E-05
**positive regulation of immune system process**	**GO:0002684**	**2.08E-05**
metal ion transport	GO:0030001	2.24E-05
signal transduction	GO:0007165	2.26E-05
terpenoid metabolic process	GO:0006721	2.31E-05
positive regulation of lipase activity	GO:0060193	2.89E-05
regulation of cell activation	GO:0050865	3.18E-05
multicellular organismal signaling	GO:0035637	3.23E-05
**regulation of immune response**	**GO:0050776**	**3.32E-05**
positive regulation of phospholipase C activity	GO:0010863	3.99E-05
retinoid metabolic process	GO:0001523	4.15E-05
regulation of hormone levels	GO:0010817	5.16E-05
phototransduction, visible light	GO:0007603	5.69E-05
activation of phospholipase C activity by G-protein coupled receptor protein signaling pathway coupled to IP3 second messenger	GO:0007200	5.77E-05
**leukocyte migration**	**GO:0050900**	**5.97E-05**
second-messenger-mediated signaling	GO:0019932	6.39E-05
cyclic nucleotide metabolic process	GO:0009187	6.49E-05
regulation of transport	GO:0051049	6.53E-05
**leukocyte differentiation**	**GO:0002521**	**7.98E-05**
actin filament-based movement	GO:0030048	8.23E-05
signal release	GO:0023061	8.31E-05
negative regulation of endopeptidase activity	GO:0010951	9.91E-05
regulation of phospholipase activity	GO:0010517	0.00010543
killing of cells of other organism	GO:0031640	0.00010739
detection of light stimulus	GO:0009583	0.000109824

cell chemotaxis	GO:0060326	0.000113183
**humoral immune response**	**GO:0006959**	**0.000122062**
hormone biosynthetic process	GO:0042446	0.000135912
protein activation cascade	GO:0072376	0.000136801
**regulation of interleukin-8 production**	**GO:0032677**	**0.000148498**
positive regulation of calcium-mediated signaling	GO:0050850	0.000148498
**leukocyte activation**	**GO:0045321**	**0.000166592**
G-protein signaling, coupled to cyclic nucleotide second messenger	GO:0007187	0.000168609
blood circulation	GO:0008015	0.000194988
**regulation of lymphocyte activation**	**GO:0051249**	**0.000195483**
protein-lipid complex remodeling	GO:0034368	0.000195734
response to chemical stimulus	GO:0042221	0.000200251
acute-phase response	GO:0006953	0.000205195
transmembrane transport	GO:0055085	0.000214325
organ morphogenesis	GO:0009887	0.000236177
induction of apoptosis by hormones	GO:0008628	0.000239587
urea transport	GO:0015840	0.000239587
